# Serum LAG-3 Predicts Outcome and Treatment Response in Hepatocellular Carcinoma Patients With Transarterial Chemoembolization

**DOI:** 10.3389/fimmu.2021.754961

**Published:** 2021-10-07

**Authors:** Mengzhou Guo, Feng Qi, Qianwen Rao, Jialei Sun, Xiaojing Du, Zhuoran Qi, Biwei Yang, Jinglin Xia

**Affiliations:** ^1^ Liver Cancer Institute, Zhongshan Hospital, Fudan University, Shanghai, China; ^2^ Department of Gastroenterology, The Shanghai Tenth People’s Hospital of Tongji University, Shanghai, China; ^3^ Minhang Hospital, Fudan University, Shanghai, China

**Keywords:** LAG-3, PD-L1, hepatocellular carcinoma, transarterial chemoembolization, tumor response

## Abstract

**Background:**

Transarterial chemoembolization (TACE) stands for the most commonly utilized therapy for hepatocellular carcinoma (HCC) worldwide. This study was to explore the potential predictive and prognostic roles of LAG-3 and PD-L1 as serum biomarkers in HCC patients underwent TACE treatment.

**Methods:**

A total of 100 HCC patients receiving TACE as well as 30 healthy controls were enrolled in the study. Serum LAG-3 and PD-L1 levels were determined at baseline and 3 day after TACE using enzyme-linked immunosorbent assay (ELISA).

**Results:**

We found serum levels of LAG-3 and PD-L1 were significantly elevated in HCC patients compared with healthy controls. Interestingly, patients with low pre-TACE and post-TACE levels of LAG-3 but not PD-L1 had a high probability of achieving an objective response (OR) after TACE treatment. Additionally, high pre-TACE LAG-3 level was correlated with poor disease outcome, and the patients with both high serum LAG-3 and PD-L1 level had the shorter overall survival (OS) than patients who are either PD-L1 or LAG-3 high or both PD-L1 and LAG-3 low. High pre-TACE serum LAG-3 level was positively associated with more cirrhosis pattern, advanced BCLC stage, pre-TACE alanine aminotransferase (ALT) level, and pre-TACE aspartate aminotransferase (AST) level. Furthermore, in 50 patients who underwent TACE, the serum LAG-3 level was significantly decreased at 3 day after TACE.

**Conclusion:**

Both pre-TACE and post-TACE serum LAG-3 levels could serve as powerful predictors for tumor response of TACE, and high pre-TACE serum LAG-3 level was an indicator for poor prognosis in HCC.

## Introduction

HCC is one of the most common malignant tumors with the fourth leading cause of cancer mortality globally (1). Due to the lack of early symptoms, roughly 70% of HCC patients are diagnosed at intermediate or advanced stage and ineligible for radical surgery, thereby leading to an unfavorable prognosis (2). TACE is one of the first-line therapeutic options for HCC patients with intermediate stage (3). In particular, according to the Chinese guideline, the application of TACE was enlarged from BCLC stage A to stage C with well-preserved liver function (4). During TACE, the transhepatic arterial infusion of both cytotoxic agent and embolic agents contributes to a dual effect of cytotoxicity and ischemia in the tumor tissues, while normal liver tissue is protected from this therapy because its main blood supply comes from the portal vein (5). Although TACE demonstrated a significant survival benefit compared with best supportive care, a large number of patients were refractory to TACE (6, 7). Therefore, it is imperative to select the optimal patients for TACE or improve the efficacy of TACE by combining with other therapies.

Immunotherapy could boost immune system by suppressing immune checkpoints, and it was regarded as a main breakthrough in the treatment paradigm for malignancies. Multiple immunomodulatory agents targeting different immune checkpoints such as programmed cell death-1 (PD-1), programmed cell death ligand-1(PD-L1) and cytotoxic T-lymphocyte antigen-4 (CTLA-4) are under investigation in various types of cancer. Both nivolumab and pembrolizumab, the anti-PD-1 monoclonal antibody, were approved as second-line treatments for advanced HCC by Food and Drug Administration (FDA) based on Checkmate 040 (8) and Keynote 224 Trials (9). However, subsequent phase 3 trials have failed to show longer OS in either first-line (nivolumab vs. sorafenib) (10) or second-line (pembrolizumab vs. placebo) setting (11). Recently, the combination of atezolizumab (anti-PD-L1 monoclonal antibody) and bevacizumab resulted in superior OS than sorafenib in the first-line treatment of patients with advanced HCC (12), which suggested that improving the efficacy of immunotherapy may depend on combined treatment. Thus, a combination of immune checkpoint inhibitors (ICIs) and TACE could be an attractive option, as TACE may regulate immune function by liberating tumor-associated antigens and enhancing tumor-specific T-cell response. Previous studies have demonstrated that the serum levels of some immunosuppressive molecules, such as T-cell immunoglobulin and mucin domain–3 (TIM-3), CTLA-4 and herpesvirus entry mediator (HVEM), were increased after TACE (13). However, the effects of TACE on other representative immune checkpoints, such as PD-L1 (13, 14) and lymphocyte activation gene 3 (LAG-3), were not settled. LAG-3, a member of immune checkpoint receptor, through the interaction with its ligand, downregulates the proliferation and activation of T-cell (15, 16). Specifically, the expression of LAG-3 on tumor infiltrating lymphocytes (17), natural killer cells (18), B cells (19) and dendritic cells (20) indicates its involvement in a widespread and complex immune pathways. LAG-3 is evaluated in various types of cancer (21–23) and has a synergistic effect with PD-1/PD-L1 axis (24–26). According to our recent study, high expression of LAG-3 in tumor tissue indicated an unfavorable prognosis in HCC (27). It is possible that targeting LAG-3 immune checkpoint could improve the efficacy of existing standard treatments, such as TACE, sorafenib and anti-PD-1/PD-L1 therapy.

Here, we aimed at evaluating the predictive and prognostic values of LAG-3 and PD-L1 as serum biomarkers for HCC patients treated with TACE, and investigating the changes of serum LAG-3 and PD-L1 levels after TACE.

## Materials And Methods

### Patients

A total of 100 patients with a confirmed diagnosis of HCC treated with TACE at Zhongshan Hospital between September 2013 and March 2021 were enrolled. The criteria of patient inclusion and exclusion were as follows: (1) no any prior anti-HCC treatment before TACE; (2) Child-Pugh classification A or B; (3) Eastern Cooperative Oncology Group Performance Status (ECOG PS) of 0-2; (4) all patients had complete clinicopathologic and follow-up information; (5) all patients had blood samples and laboratory data at baseline and post-TACE time, such as alanine transaminase, alanine aminotransferase and bilirubin; (6) no history of other types of cancer. In addition, a total of 30 healthy populations with normal liver function were enrolled as negative controls. Informed consents were obtained from every individual and the study was approved by the local ethics committee of Zhongshan Hospital.

### Sample Collection and Measurement of Serum LAG-3 and PD-L1 Levels

Serum samples were obtained from all patients at baseline (1 or 2 days before TACE) and 3 day after TACE. The serum tubes were centrifuged at 3000 rpm for 10 min at 4^◦^C and then aliquoted and stored at −80^◦^C. Serum LAG-3 concentrations were measured by ELISA using the Human LAG-3 ELISA Kit (Cat: BMS2211, Thermo Fisher Scientific Waltham, MA, USA), according to the manufacturer’s instructions. Serum PD-L1 was determined by the Human PDL1 ELISA Kit (Cat: SU-B12533, China) according to the recommendation of the manufacturer.

### TACE Treatment and Evaluation of Treatment Response

The TACE was performed as the standard modality of the institution (28). Briefly, the aimed tumor feeding arteries were catheterized with a 4-Fr or 5-Fr RH catheter or combined with microcatheters if necessary. Then, an emulsion of oxaliplatin (100–150 mg), 5-fluorouracil (500-1000mg) and lipiodol (5-20ml) was injected into the target vessels. For some patients with arterioportal shunt or prominent hypervascularity, gelatin sponge particles were selected. Two experienced radiologists performed the tumor measurements. Tumor response was evaluated based on radiological assessment (CT or MRI) according to the modified Response Evaluation Criteria in Solid Tumors (mRECIST), including complete remission (CR), partial remission (PR), stable disease (SD), and progression disease (PD). CR and PR were defined as OR, and SD or PD were summarized into non-objective response (non-OR).

### Statistical Analysis

All statistical analyses were performed with SPSS 23.0 software (IBM, USA) and Graphs were created using the GraphPad Prism version 7.0 software. Continuous variables were compared using Mann–Whitney U test and Wilcoxon Rank-Sum test. Categorical variables were analyzed using the Pearson chi-squared test or Fisher’s exact test (if any expected value < 5 was found). The predictive values of serum LAG-3 and PD-L1 were evaluated by the receiver operator characteristic (ROC) curve analysis. The optimal cutoffs of variables for estimating sensitivity and specificity were established using the maximum Youden’s index. OS was calculated from the date of diagnosis to the date of death or the last known follow-up. The survival curves were estimated using the Kaplan–Meier method and survival rates compared by the log-rank test. Multivariate analysis was performed by Cox regression to evaluate the independent prognostic factors. A two-sided *P* value < 0.05 was considered statistically significant.

## Results

### Baseline Characteristics of HCC Patients Enrolled

Overall, 100 patients with HCC were enrolled in this study. Baseline characteristics are presented in **Table 1**. The median patient age was 58 years, and 86% of the patients were men. Most patients had cirrhosis (86%), and 86% were determined to be HBsAg positivity. According to the Barcelona Clinic Liver Cancer (BCLC) criteria, 19%, 43%, and 38% of the patients were stage A, B, and C, respectively.

**Table 1:** Characteristics of 100 patients with hepatocellular carcinoma treated with transarterial chemoembolization.

Variables	Median (range) or number (%)
Age (years)	58 (23-82)
Gender	
Male	86 (86%)
Female	14 (14%)
BCLC stage	
A	19 (19%)
B	43 (43%)
C	38 (38%)
HBsAg	
Positive	86 (86%)
Negative	14 (14%)
Tumor size (cm)	
>5	57 (57%)
≤5	43 (43%)
Tumor number	
Single	42 (42%)
Multiple	58 (58%)
Extrahepatic metastasis	
Yes	12 (12%)
No	88 (88%)
Portal vein invasion	
Yes	32 (32%)
No	68 (68%)
Cirrhosis	
Yes	86 (86%)
No	14 (14%)

HBsAg, hepatitis B surface antigen; AFP, αfetoprotein; BCLC, Barcelona Clinic Liver Cancer.

### Baseline Serum Levels of LAG-3 and PD-L1 Were Elevated in HCC

**Figure 1:**
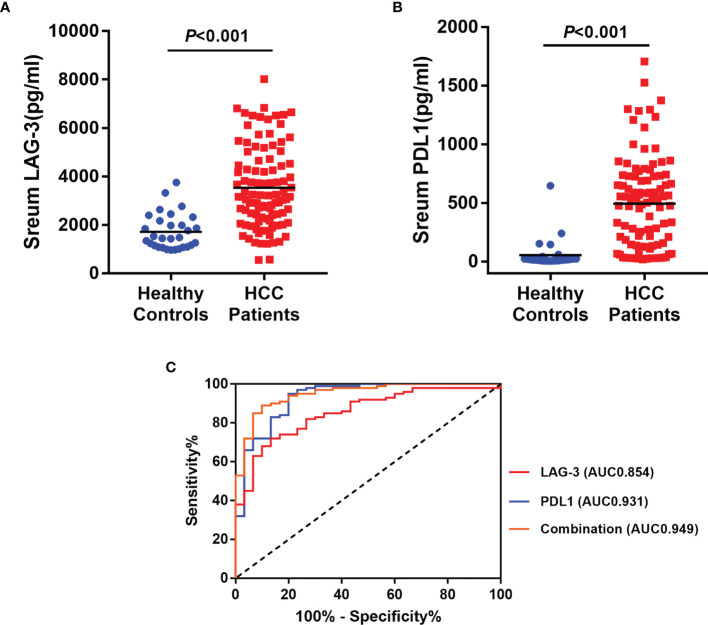
Diagnostic significances of serum LAG-3 and PD-L1 in HCC patients underwent TACE. **(A)** Serum LAG-3 level was elevated in patients underwent TACE. **(B)** Serum PD-L1 level was elevated in patients underwent TACE. **(C)** ROC curve analysis of LAG-3, PD-L1 and combination of LAG-3 and PD-L1 differentiating HCC patients from healthy controls. HCC, hepatocellular carcinoma; TACE, Transarterial chemoembolization; ROC, receiver operating characteristics; AUC, area under curve.

Baseline serum levels of LAG-3 and PD-L1 were both elevated in HCC patients than healthy controls (**Figures 1A, B**). Next, ROC curve was used to evaluate the diagnostic performance of LAG-3 and PD-L1 in differentiating HCC from healthy controls. As shown in **Figure 1C**, the area under the curve (AUC) of LAG-3 and PD-L1 were 0.854 (95%CI: 0.784-0.925) with 72.0% sensitivity and 86.7% specificity, 0.931 (95%CI: 0.876-0.987) with 95.0% sensitivity and 80.0% specificity, respectively. Moreover, the combination of the serum LAG-3 and PD-L1 generated an increased diagnostic AUC value of 0.949 (95% CI: 0.908-0.990) with 89% sensitivity and 90% specificity, which is better than the LAG-3 or PD-L1 alone.

### Correlation Between Pre-TACE Serum LAG-3 and PD-L1 and Response to Treatment

**Figure 2:**
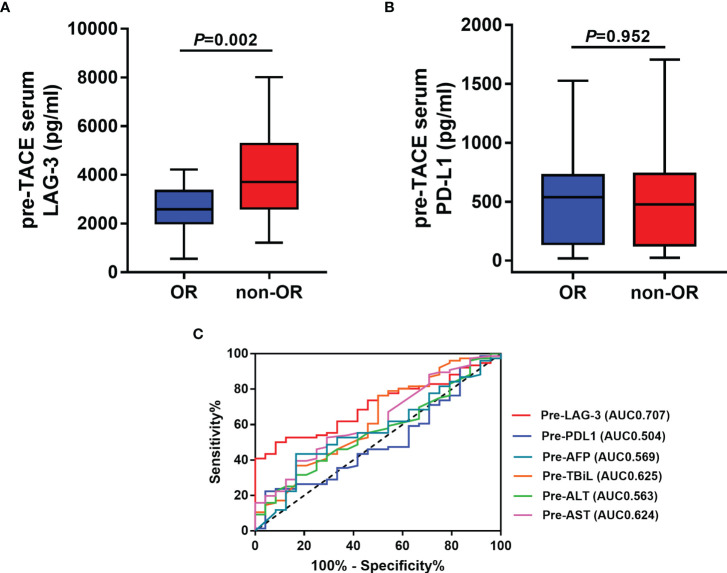
Pre-TACE LAG-3 level as a serum indicator for predicting OR after TACE. **(A)** Pre-TACE serum levels of LAG-3 were significantly lower in patients who showed an OR to TACE compared to non-OR patients; **(B)** Pre-TACE serum levels of PD-L1 were unaltered between OR and non-OR patients; **(C)** ROC curve analysis of different variables for the discrimination between OR and non-OR patients. OR, objective response; non-OR, non-objective response.

We next assessed whether the pretreatment serum LAG-3 and PD-L1 levels could predict tumor response to TACE therapy in HCC. Therefore, our cohort was divided into patients with an OR (n=24) or non-OR (n=76) after TACE. In the analysis, the pretreatment serum LAG-3 level was significantly lower in patients with an OR compared to that in patients who showed a non-OR (**Figure 2A**), while the pretreatment serum levels of PD-L1 did not significantly change between the two groups (**Figure 2B**). Subsequently, we performed ROC curves to identify the efficacy of serum LAG-3 and PD-L1 for predicting tumor response. The AUC of pre-TACE serum LAG-3 levels was 0.707, which was superior to pre-TACE serum PD-L1 levels (AUC = 0.504) (**Figure 2C**). We also compared the predictive values of some clinical parameters with serum LAG-3 and PD-L1. The results showed that the AUCs of serum αfetoprotein (AFP), total bilirubin (TBiL), ALT and AST were 0.569,0.625,0.563 and 0.624, respectively, which were worse than the predictive value of pretreatment serum LAG-3 in differentiating OR from non-OR (**Figure 2C**). Moreover, using ROC curve to determine the value of serum LAG-3 for predicting tumor response to TACE therapy, we chose >3723.1pg/ml as the cut-off point that combined maximal sensitivity with best specificity. Therefore, this cohort was classified as low LAG-3 or high LAG-3 group based on the cut-off value for subsequent investigations. The cut-off values of other indicators were showed in **Table 2**.

**Table 2:** Discriminant abilities and the optimal cut-off values of the variables examined.

Variables	AUC	*P* value	HR (95%CI)	Sensitivity (%)	Specificity (%)	Cut-off value
Pre-TACE						
LAG-3	0.707	0.002	0.605-0.809	50	91.7	3723.06 pg/ml
PD-L1	0.504	0.952	0.374-0.635	22.4	95.8	792.27 pg/ml
AFP	0.569	0.307	0.443-0.696	43.4	83.3	843.60 ng/ml
TBiL	0.625	0.066	0.494-0.756	76.3	50.0	10.40μmol/L
ALT	0.563	0.355	0.438-0.688	31.6	83.3	55.50 U/L
AST	0.624	0.068	0.500-0.748	52.6	70.8	45.50 U/L
Post-TACE						
LAG-3	0.751	0.020	0.596-0.905	46.3	100	2194.30 pg/ml
PD-L1	0.569	0.520	0.384-0.755	24.4	100	977.79 pg/ml

AFP, αfetoprotein; TBiL, total bilirubin; ALT, alanine aminotransferase; AST, aspartate aminotransferase; HR, hazard ratio; CI, confidential interval. P < 0.05 was considered statistically significant.

### Associations Between Pre-TACE Serum LAG-3 and PD-L1 Levels and Clinical Characteristics of HCC Patients

The associations between pre-TACE serum levels of LAG-3 and PD-L1 and clinicopathologic parameters were summarized in **Table 3**. High pre-TACE serum LAG-3 level was found to correlate with a more cirrhosis pattern (*P*= 0.034), high pre-TACE AST levels (*P*=0.020), high pre-TACE ALT levels (*P*=0.029), and advanced BCLC stage (*P*=0.017). Furthermore, high pre-TACE serum PD-L1 level correlated positively with high pre-TACE TBiL levels (*P*=0.012).

**Table 3:** The association between the serum LAG-3 and PD-L1 level with clinical characteristics of HCC patients.

Variable	LAG-3			PD-L1		
	Low	High	*P*	Low	High	*P*
Patients	60	40		82	18	
Age(years)			0.927			0.707
≤ 50	16	11		21	6	
> 50	44	29		61	12	
Gender			0.724			1.000
Female	9	5		11	3	
Male	51	35		71	15	
HbsAg			0.158			0.444
Negative	6	8		13	1	
Positive	54	32		69	17	
Cirrhosis			0.034			0.988
No	12	2		12	2	
Yes	48	38		70	16	
Portal vein invasion			0.161			0.893
Absent	44	24		56	12	
Present	16	16		26	6	
Tumor size, cm			0.187			0.697
≤5	29	14		36	7	
>5	31	26		46	11	
Tumor number			0.934			0.817
Single	25	17		34	8	
Multiple	35	23		48	10	
AFP(ng/ml)			0.146			0.578
≤20	20	8		22	6	
>20	40	32		60	12	
Pre-TACE TB,μmol/L			0.373			0.012
≤10.4	20	10		29	1	
>10.4	40	30		53	17	
Pre-TACE ALT,U/L			0.029			0.578
≤55.5	48	24		60	12	
>55.5	12	16		22	6	
Pre-TACE AST,U/L			0.020			0.166
≤48.5	41	18		51	8	
>48.5	19	22		31	10	
BCLC stage			0.017			0.958
0+A	16	3		15	4	
B+C	44	37		67	14	

HBsAg, hepatitis B surface antigen; AFP, αfetoprotein; TBiL, total bilirubin; ALT, alanine aminotransferase; AST, aspartate aminotransferase; BCLC, Barcelona Clinic Liver Cancer. P < 0.05 was considered statistically significant.

### Prognostic Significances of Pre-TACE Serum LAG-3 and PD-L1 Levels in HCC

**Figure 3:**
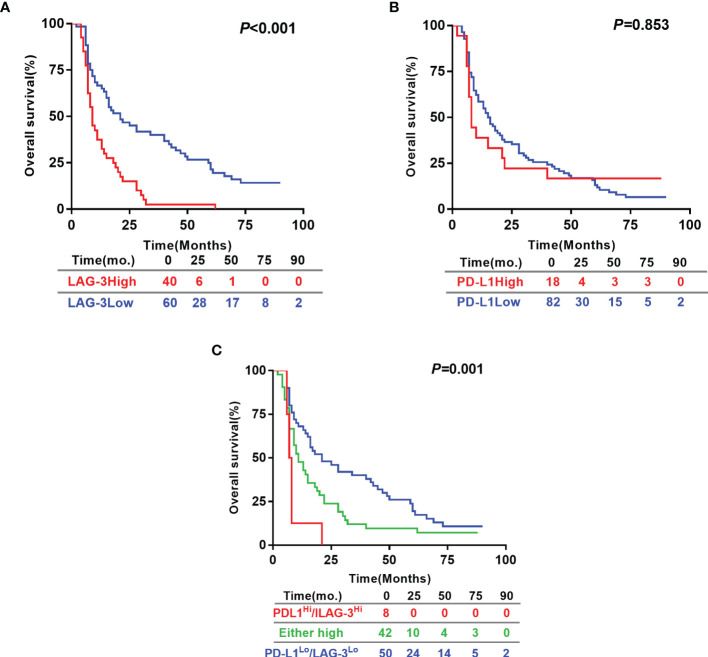
Prognostic significance of pre-TACE serum LAG-3 and PD-L1 levels in HCC underwent TACE. Kaplan–Meier analysis of OS based on the serum LAG-3 level **(A)**, serum PD-L1 level **(B)** and the combination of the serum levels of LAG-3 and PD-L1 **(C)**. Differences between groups were evaluated using log-rank test. The number of patients at risk was reported. OS, Overall survival.

Based on the exciting finding on the powerful value of LAG-3 as serum biomarker for predicting tumor response to TACE therapy, we continued to evaluate the prognostic significances of pretreatment serum levels of LAG-3 and PD-L1. For the 100 HCC patients, the 5 year OS rate was 15%. The patients with high pre-TACE serum LAG-3 level (>3723.06 pg/ml) had significantly shorter OS (13.63 months vs. 34.43 months, *P <*0.001) than patients with low serum LAG-3 level (≤3723.06 pg/ml) (**Figure 3A**). However, OS did not statistically correlate with pre-TACE serum PD-L1 level (**Figure 3B**). We next performed a multivariate cox regression analysis to substantiate the prognostic value of serum LAG-3 level in this setting. The parameters with *P* values < 0.05 using Kaplan-Meier analysis were entered into the multivariate cox proportional hazards analysis. Multivariate analysis revealed that pre-TACE serum LAG-3 level, portal vein invasion and pre-TACE serum AFP level were independent prognostic factors for OS (**Table 4**). Although PD-L1 expression was not significantly associated with OS, we still wondered whether combining PD-L1 and LAG-3 would influence prognosis based on the finding that LAG-3 had synergistic effects with PD-1/PD-L1. Our data suggested that patients with high expression of both LAG-3 and PD-L1 showed shorter OS than patients who are either PD-L1 or LAG-3 high or both low expression of PD-L1 and LAG-3 (**Figure 3C**).

**Table 4:** Univariate and multivariate analysis for OS.

Variables	Univariate	Multivariate	
	*P* value	HR(95% CI)	*P* value
Age, years (>50 vs.≤50)	0.530		NA
Gender (male vs. female)	0.879		NA
HBsAg (positive vs. negative)	0.373		NA
AFP, ng/ml (>20 vs. ≤20)	0.001	1.890 (1.120-3.189)	0.017
Cirrhosis (yes vs. no)	0.610		NA
Tumor number (multiple vs. single)	0.393		NA
Tumor size, cm (>5 vs. ≤5)	0.000		NS
Metastasis (yes vs. no)	0.030		NS
Portal vein invasion (present vs. absent)	0.000	2.786 (1.550-5.008)	0.001
Pretreatment serum LAG-3 (high vs. low)	0.000	2.076 (1.273-3.385)	0.003
Pretreatment serum PD-L1 (high vs. low)	0.853		NA
Baseline bilirubin (high vs. low)	0.716		NA
Baseline ALT (high vs. low)	0.066		NA
Baseline AST (high vs. low)	0.001		NS

OS, overall survival; DFS, disease-free survival; HBsAg, hepatitis B surface antigen; AFP, αfetoprotein; HR, hazard ratio; CI, confidential interval; NA, not adopted; NS, not significant. P < 0.05 was considered as statistically significant.

### The Changes of Serum LAG-3 and PD-L1 Levels After TACE Treatment, and Determination of the Predictive and Prognostic Values of Post-TACE Serum Levels of LAG-3 and PD-L1 for 50 Patients

**Figure 4:**
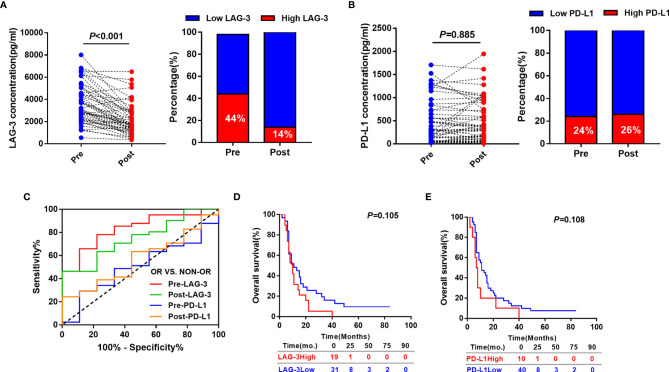
Predictive and prognostic values of post-TACE serum LAG-3 and PD-L1 levels in HCC patients underwent TACE. **(A)** Distribution of serum LAG-3 level (left) and proportion of high LAG-3 (right) in HCC patients before and after TACE. **(B)** Distribution of serum PD-L1 level (left) and proportion of high PD-L1 (right) in HCC patients before and after TACE. **(C)** ROC curve analysis of pre-TACE and post-TACE serum LAG-3 and PD-L1 level for the discrimination between OR and non-OR patients. **(D)** Kaplan-Meier curve of OS according to post-TACE LAG-3 level in HCC patients received TACE. **(E)** Kaplan-Meier curve of OS according to post-TACE PD-L1 level in HCC patients received TACE.

Out of 100 patients, serum samples at 3 day after TACE of 50 patients who met the inclusion criteria were collected. We subsequently determined whether post-TACE serum LAG-3 and PD-L1 levels might also serve as a biomarkers for predicting tumor response to TACE. Median serum LAG-3 level decreased significantly at the 3 day post-TACE compared with pre-TACE (Median: 1725.77pg/ml vs.3188.23pg/ml, P<0.001) (**Figure 4A**), while serum levels of PD-L1 showed no significant difference (Median: 508.21pg/ml vs. 358.56pg/ml, *P*=0.885) (**Figure 4B**). In line with this, the percentage of patients with high LAG-3 was reduced after TACE treatment (44% vs.14%) (**Figure 4A**), and no significant difference was observed in the percentage of patients with high PD-L1 between the days of pre-TACE and 3-day post-TACE (24% vs.26%) (**Figure 4B**).

We subsequently evaluated if post-TACE serum LAG-3 level might also has a value for predicting tumor response to TACE. As expected, the post-TACE serum LAG-3 level was significantly decreased in responsive HCC patients compared to nonresponsive patients (*P* = 0.020), whereas the post-TACE serum PD-L1 level did not significantly change (*P* = 0.520). Next, Using ROC curve analysis, post-TACE LAG-3 was found to show an increased predictive AUC value of 0.751 (95% CI = 0.596–0.905), which is better than the AUC value of post-TACE PD-L1(AUC:0.569, 95% CI = 0.384–0.755). Furthermore, there were no significant differences in AUC values of serum LAG-3 and PD-L1 levels between pretreatment and posttreatment time (**Figure 4C**).

Finally, we assessed if serum levels of post-TACE LAG-3 and PD-L1 could predict the patients’ prognosis in this cohort. The optimal cut-off points were determined by the maximum Youden’s index using ROC analysis. Using the stratification cut-off points as in the assessment of tumor response, the patients with high post-TACE concentrations of LAG-3 and PD-L1 were not correlated with a significantly but a tendency towards impaired OS (**Figures 4D, E**).

## Discussion

As previously mentioned, the patients who will likely benefit from TACE treatment is not fully understood. The-TACE induced tumor necrosis and local hypoxia may result in adaptive changes in the expression of proangiogenic cytokines, hypoxia-related elements and immune factors in the tumor microenvironment. Recently, the application of ICIs has led to a clinical breakthrough for HCC, and made efforts to improve the efficacy of TACE (29). Thus, combination treatment of TACE and ICIs may generate promising outcome in TACE-refractory HCC. Subsequently, an increasing number of studies have investigated the effects of TACE on immune function in HCC. The proportions of T-regulatory cell were found to be reduced after TACE, which indicated that TACE could restore immune function (30). Furthermore, other studies reported that TACE provokes a significant increase in the expression of some immune checkpoints, such as TIM-3、PD-1、PD-L1 and CTLA4, which have been explained by a reactive expansion as a negative feedback mechanism in response to intense immune stimulation following tumor necrosis (13). However, there were some contradictory findings on the predictive values of immune checkpoints for tumor response to TACE. Tampaki reported that complete responders had higher serum TIM-3 levels than partial responders after TACE (31), whereas the expression of PD-L1 and PD-1 were significantly lower in patients with well TACE response than those patients with poor response to TACE (32). In brief, it is of great significance to figure out the regulatory effects of TACE on immune function. LAG-3 is an emerging immune checkpoint molecule that represses the proliferation and effector response of T cells. Due to the immune suppressive function similar to PD-1 and PD-L1, LAG-3 overexpression on tumor tissue has been reported to be associated with unfavorable clinical parameters and poor prognosis in various types of cancer, such as melanoma (24), head and neck squamous cell carcinoma (21), non-small cell lung cancer (NSCLC) (26), diffuse large B- cell lymphoma (33) and so on. Previously we reported that high tumor tissue expression of LAG-3 was correlated with poor outcome in HCC patients, whereas PD-L1 showed no significant correlation with prognosis (27). Our study indicated that LAG-3 may play a more crucial role in the development of immunosuppression against HCC when compared with the PD-1/PD-L1 axis. However, the role of serum LAG-3 in caner has not been settled. In breast (34) and gastric cancer (35), high levels of serum LAG-3 were correlated with improved prognosis. In NSCLC, low serum LAG-3 expression was associated with advanced or metastatic disease (36). On the contrary, high levels of soluble LAG-3 were association with poor PFS and OS in advanced head and neck cancer (37). These data suggested that further investigation of the prognostic and predictive roles of serum LAG-3 in other types of cancer is required. To our knowledge, this study is the first to investigate the predictive and prognostic values of both LAG-3 and PD-L1 as serum biomarkers in HCC patients underwent TACE, and evaluate the dynamic changes of serum LAG-3 and PD-L1 levels in the context of TACE treatment.

In the present study, the responsive patients had a lower pre-TACE levels of LAG-3 than non-responsive patients, whereas pre-TACE PD-L1 levels showed no significant difference between OR and non-OR patients. Similar to our result about LAG-3, the pre-TACE PD-L1 and PD1 expression were significant lower in patients with well TACE response than poor TACE response group (32), and the paradoxical result about PD-L1 may attribute to the differences in patient cohorts. Generally speaking, high levels of LAG-3 were positively correlated with large tumor burden and immune tolerance, which may contribute to less effective of TACE. Using the stratification cut-off points as in the assessment of tumor response, we found that high levels of LAG-3 were positively correlated with unfavorable clinical parameters, such as advanced tumor stage, more cirrhosis pattern and degree of hepatic damage. Moreover, patients with high serum levels of LAG-3 showed significantly shorter OS than patients with low levels of LAG-3, and further multivariate analysis confirmed that pre-TACE LAG-3 level was an independent indicator for OS. The similar results have been reported in various types of cancer (38). Although PD-L1 expression was not significantly associated with OS, we still wondered whether combining PD-L1 and LAG-3 would influence prognosis based on the finding that LAG-3 had synergistic effects with PD-1/PD-L1. Our data suggested that patients with high expression of both LAG-3 and PD-L1 showed shorter OS than patients who are either PD-L1 or LAG-3 high or both low expression of PD-L1 and LAG-3. These results confirm to the findings that blockade both LAG-3 and PD-L1 pathways showed a greater therapeutic effect than blockade of either alone *in vivo* researches (39, 40).

Except for pre-TACE immune molecules, we were also concerned about the predictive and prognostic roles of post-TACE serum LAG-3 and PD-L1 levels. It was found that the serum levels of LAG-3 were decreased significantly at the day 3 after TACE treatment. As mentioned, LAG-3 expression is upregulated by cytokines such as IL-2 and IL-12 (41), and positively correlates with IFN-γ production (42). Previous studies indicated that some cytokine profiles were decreased by day 3 after TACE therapy, such as IL-2, IL-12 and IFN-γ (43). Therefore, a possible explanation for a decreased pattern of LAG-3 may attribute to the accompanying reduction of some cytokines. As we expected, post-TACE serum LAG-3 level was able to predict the tumor response to TACE therapy, while the PD-L1 was not. This finding may demonstrated that the LAG-3 related pathway was very promising in HCC patients treated with TACE, and LAG-3 blockade had the potential to enhance the antitumor effect of TACE.

There are some limitations of this study. First, this is a retrospective study with a small sample size, which affects the power of statistical test. Second, we only test serum samples at day 3 after TACE. To elucidate dynamic changes of immune molecules in the context of TACE, plasma samples at more time points should be collected. Third, the expression of other immune molecules should be detected together with the LAG-3 and PD-L1 to better understand the mechanism of TACE therapy affecting tumor immunity, so as to provide theoretical basis for the combination of TACE and immunotherapy in HCC patients.

## Data Availability Statement

The original contributions presented in the study are included in the article/**Supplementary Material**. Further inquiries can be directed to the corresponding authors.

## Ethics Statement

The studies involving human participants were reviewed and approved by Ethics Committee of Zhongshan Hospital. The patients/participants provided their written informed consent to participate in this study.

## Author Contributions

MG, BY, and JX designed the study, performed the experiment and wrote the manuscript. FQ and QR analyzed the data and performed figures and tables. JS, XD, and ZQ contributed to patient follow-up. All authors contributed to the article and approved the submitted version.

## Funding

This study was supported by the National Natural Science Foundation of China (grant no. 81772590 and 81572395).

## Conflict of Interest

The authors declare that the research was conducted in the absence of any commercial or financial relationships that could be construed as a potential conflict of interest.

## Publisher’s Note

All claims expressed in this article are solely those of the authors and do not necessarily represent those of their affiliated organizations, or those of the publisher, the editors and the reviewers. Any product that may be evaluated in this article, or claim that may be made by its manufacturer, is not guaranteed or endorsed by the publisher.
